# High Photoelectric Conversion Efficiency of Metal Phthalocyanine/Fullerene Heterojunction Photovoltaic Device

**DOI:** 10.3390/ijms12010476

**Published:** 2011-01-17

**Authors:** Chi-Feng Lin, Mi Zhang, Shun-Wei Liu, Tien-Lung Chiu, Jiun-Haw Lee

**Affiliations:** 1 Graduate Institute of Photonics and Optoelectronics, National Taiwan University, No. 1, Section 4, Roosevelt Road, Taipei 10617, Taiwan; E-Mails: f94941089@ntu.edu.tw (C.-F.L.); jiunhawlee@ntu.edu.tw (J.-H.L.); 2 Department of Photonics Engineering, Yuan Ze University, Taoyuan 32003, Taiwan; E-Mail: vzenazhang@gmail.com; 3 Institute of Chemistry, Academia Sinica, No. 128, Academia Road, Section 2, Nankang, Taipei 115, Taiwan

**Keywords:** OPV, energy conversion efficiency, heterojunction

## Abstract

This paper introduces the fundamental physical characteristics of organic photovoltaic (OPV) devices. Photoelectric conversion efficiency is crucial to the evaluation of quality in OPV devices, and enhancing efficiency has been spurring on researchers to seek alternatives to this problem. In this paper, we focus on organic photovoltaic (OPV) devices and review several approaches to enhance the energy conversion efficiency of small molecular heterojunction OPV devices based on an optimal metal-phthalocyanine/fullerene (C_60_) planar heterojunction thin film structure. For the sake of discussion, these mechanisms have been divided into electrical and optical sections: (1) Electrical: Modification on electrodes or active regions to benefit carrier injection, charge transport and exciton dissociation; (2) Optical: Optional architectures or infilling to promote photon confinement and enhance absorption.

## 1. Introduction

Since the industrial revolution in the 19th century, the demand for energy has been growing rapidly, giving rise to the development of fossil fuels (coal, petroleum and gas), and nuclear energy. Issues related to the limited supply of natural resources and global warming from ejective pollution, have prompted scientists to invest a great deal of effort in seeking a clean and unfailing supply of alternative energy for future generations. Without doubt, solar energy shows promise as a green energy, because it is non-polluting, inexhaustible and a renewable energy source [1]. One device used to convert solar energy to electrical power is called a photovoltaic device. Several types of efficient and mature photovoltaic devices can be distinguished according to their content and structure, with characteristics such as inorganic base: Silicon, III–V and II–VI PN junction photovoltaic device [2,3], Copper-Indium-Gallium-Selenium (CIGS) thin film photovoltaic device [2]; organic base: dye sensitizer photovoltaic device [4] and organic thin film photovoltaic device [5].

Currently, inorganic photovoltaic devices perform with higher photoelectric conversion efficiency (PCE) and stability, than organic photovoltaic devices. However, inorganic photovoltaic devices still have deficiencies, such as high manufacturing cost and solid construction, which hampers their application as cheap consumables and flexible electronic products. The development of organic photovoltaic (OPV) devices may play a key role in overcoming the deficiencies of inorganic photovoltaic devices, because they offer several advantages such as: Lower energy and material consumption during the manufacturing process, low cost, low temperature process compatible with flexible substrates, and extremely lightweight [5–7]. Hence, OPV devices with these significant advantages have attracted a great deal of attention, forcing researchers to invest a great deal of effort in pursuing higher PCE.

The first breakthrough in the efficiency of OPV devices (1%) was disclosed by C.-W. Tang in 1985 [5]. Since then, the PCE of OPV devices has been improving steadily through the utilization of new concepts including the bulk heterojunction [6], laminated donor/accepter heterojunctions [8], exciton-blocking layer (EBL) [9], organic dopants [10], metal nanoparticle dopants, [11] stacked tandem structures [12], and p-i-n architecture [13]. So far, Chen *et al.* have proposed the most efficient polymer based OPV device with a maximum PCE of close to 8% [14], by using a bulk heterojunction structure. For small molecular OPV, Chan *et al.* reported the highest PCE of 5.58% by doping rubrene with copper phthalocyanine (CuPc) [10]. There have been many efforts to understand the physical mechanism of OPV devices and further improve device performance. Until now, small molecule OPV devices still perform at a lower efficiency than polymer OPV devices, due to the limitations of the materials. However, the development of small molecule OPV devices appears to be continuously expanding due to the ease with which film thickness can be controlled during device fabrication, as well as the excellent stability of the donor material such as CuPc and Zinc phthalocyanine (ZnPc).

Currently, improvements in the PCE of planar heterojunction small molecule OPV devices are evolving. Here, we summarize the on-going investigations from the last five years, regarding the high PCE OPV device based on a metal-phthalocyanine/fullerene (C_60_) active layer listed in Table 1. We have classified them according to modifications such as anode pretreatment [15,16], multiple junctions of active layers [17–25], as well as the material of the anode [26,27], donor [25,28–30], acceptor [31,32], and EBL [33–37]. For example, Peumans *et al.* replaced the perylene derivatives with C_60_ and introduced bathocuproine (BCP) as the EBL to achieve a PCE of 3.6% [31]. Mutolo *et al.* used subphthalocyanine (SubPc) as the donor material to obtain a PCE of 2.1% with a high open circuit voltage of 0.97 V [28]. Too numerous to mention details here, a great many notable reports are referred to in Table 1, and will be addressed in the following.

In this review, we first introduce the operational principles of OPV devices in Section 2. In Section 3, we describe the experiment including device fabrication and measurement of electro-optical characteristics. In Section 4, several modifications, designs, and improvements on photon absorption, carrier injection, and transport for high efficiency OPV devices will be discussed. Finally, a summary will be presented in Section 5.

## 2. Principle of OPV

**Figure 1:**
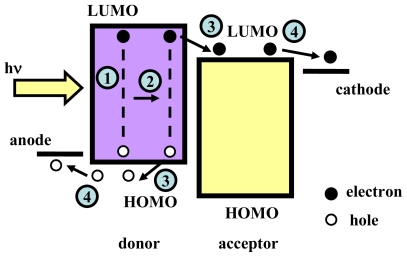
Operation process of the planar heterojunction OPV devices, the complete process including: (**1**) Photon absorption and exciton generation; (**2**) exciton diffusion; (**3**) exciton dissociation; (**4**) carriers collection.

The fundamental principle underlying planar heterojunction OPV devices and the mechanism of how photons are transformed into photocurrent are illustrated in Figure 1. There are four steps in converting photons to free carriers: Exciton generation, diffusion, and dissociation, as well as carrier collection. First, the incidental photons are absorbed by the active material within the OPV device and converted to excitons. The diffusion motion of these neutral photon-generated excitons is driven by spatially non-uniform accumulation in organic material. As long as the energy offset of the lowest occupied molecular orbital (LUMO) and the highest occupied molecular orbital (HOMO) between the donor and acceptor is large enough, excitons will be sufficiently dissociated into electron-hole pairs with a difference in binding energy near the donor/acceptor interface due to the preferable properties of charged carriers with regard to energy [38–41]. These electron-hole pairs are separated into free carriers under the assistance of a built-in electrical field [39], relative to the difference in work function between the anode and cathode. Finally, most of the free carriers drift to the electrode through the organic material under this built-in electrical field and is collected by the external circuit, thereby producing a photocurrent.

According to the description of the above four steps, one could define external quantum efficiency (EQE) as the ratio of the number of photo-generated carriers collected by the electrodes to the number of the photons incidental to the device, which could be represented as follows [38]:

(1) $$\eta_{\text{ext}}=\eta_{\text{A}}\times \eta_{\text{ED}}\times \eta_{\text{CT}}\times \eta_{\text{CC}}$$

where η_A_ is efficiency of exciton generation dependant on absorption, defined as the ratio of the number of photo-induced excitons to the number of incidental photons. η_ED_ is the exciton diffusion efficiency, defined as the ratio of the number of diffuse excitons to the donor/acceptor interface to the number of photo-induced excitons. The charge transfer efficiency, η_CT_, is defined as the probability of excitons reaching the interface and dissociating into bound electron-hole pairs. Carrier collection efficiency; η_CC_, refers to the percentage of bound electron-hole pairs that could be separated as free carriers to be collected by the electrodes. These four factors dominate the performance of OPV devices. They can be improved by replacing the material within the devices or the architectural design of the units. The refined criteria for each factor: η_A_ is dependent on the number of incidental photons and the absorption ability of the active materials. η_ED_ is relative to the distance between the heterojunction interface and the position of major photon generation, referred to as the optical field distribution within the device. η_CT_ is a minor factor and is assumed to be 100% when the energy offset of LUMO (HOMO) between donor and acceptor is larger than 0.2 eV [42]. η_CC_ depends mainly on the carrier transport affected by the mobility of the organic material and the built-in electrical field of the device. Moreover, traps, defects, and charge imbalance eliminate the number of transporting carriers, because they may be hindered or recombined.

**Figure 2:**
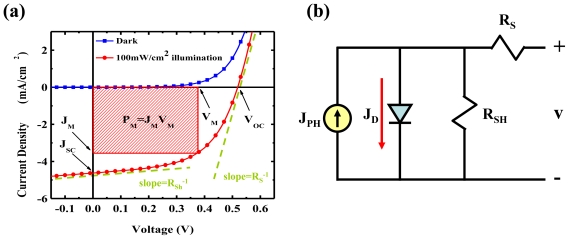
(**a**) Typical *J*-*V* characteristic of a PV device in dark condition and under illumination; (**b**) Equivalent circuit model of the PV device, which consists of a photocurrent source, a Shockley or p-n junction diode which present the *J*_D_, *R*_S_, and *R*_SH_.

Another popular parameter expressed with regard to efficiency in the field of photovoltaic devices is PCE, η_P_. Different from external quantum efficiency, PCE is defined as the percentage of light energy converted into electrical energy, and can be calculated from the current density *versus* voltage (*J*-*V*) performance of the photovoltaic device under illumination. The typical *J*-*V* curves of a photovoltaic device operated in the dark, and under illumination with its equivalent circuit, are shown in Figure 2(a) and (b) respectively. In Figure 2(a), the photovoltaic device has *J*-*V* characteristics similar to those of a Shockley diode in the dark. Under illumination, the *J*-*V* curve across the fourth quadrant indicates the major operating region and several device parameters including open-circuit voltage (*V*_OC_), short circuit current (*J*_SC_), and the point of the voltage and current that produce the maximum electrical power (*J*_M_, *V*_M_, and *P*_MAX_). Hence, the PCE can be calculated as:

(2) $$\text{PCE},\;\eta_{\text{P}}\equiv \frac{P_{\text{MAX}}}{P_{\text{Light}}}=\frac{J_{\text{M}}\cdot V_{\text{M}}}{P_{\text{Light}}}=\frac{J_{\text{SC}}\cdot V_{\text{OC}}\cdot FF}{P_{\text{Light}}}$$

where *P*_Light_ is the power density of the incidental light, the fill factor (*FF*) is defined as the ratio of the maximum actual electrical power (*J*_M_ × *V*_M_) to the maximum theoretical electrical power output (*J*_SC_ × *V*_OC_).

An equivalent circuit model is generally used to describe the electrical performance of photovoltaic devices, as shown in Figure 2(b). The equivalent circuit comprises a photocurrent source (*J*_PH_), a Shockley or p-n junction diode which present the dark current (*J*_D_), the series resistance (*R*_S_), and the shunt resistance (*R*_SH_). The *R*_S_ expresses the integral conductivity of the OPV device directly related to its internal carrier mobility. The *R*_SH_ refers to the loss of photocurrent caused to carrier recombination within the device, particularly at the interfaces of each layer. The *J*-*V* characteristic in Figure 2(a) can be analyzed by the generalized Shockley equation corresponding to this equivalent circuit [43]:

(3) $$J=\frac{1}{1+R_{\text{S}}/R_{\text{SH}}}\{J_{\text{S}}[\text{exp}(\frac{V-{JR}_{\text{S}}}{{nk}_{\text{B}}T/q})-1]-(J_{\text{PH}}-\frac{V}{R_{\text{SH}}})\}$$

where *J*_S_ and *n* are the reverse saturation current density and the ideal factor of the diode, respectively; *k*_B_ is Boltzmann’s constant; and *T* is the absolute temperature. By setting *J* = 0 and *V* = 0, the open-circuit and the short-circuit current density can also be calculated from this current equation, and can be presented as:

(4) $$V_{\text{OC}}=\frac{{nk}_{\text{B}}T}{q}\text{ln}[1+\frac{J_{\text{PH}}}{J_{\text{S}}}(1-\frac{V_{\text{OC}}}{J_{\text{PH}}R_{\text{SH}}})]$$

(5) $$J_{\text{SC}}=-\frac{1}{1+R_{\text{S}}/R_{\text{SH}}}\{J_{\text{PH}}-J_{\text{S}}[\text{exp}(\frac{|J_{\text{SC}}|R_{\text{S}}}{{nk}_{\text{B}}T/q})-1]\}$$

## 3. Experimental: Device Fabrication and Measurement

Small molecule thin films are usually grown in solvent-free processes, such as thermal sublimated deposition [44], organic vapor phase deposition (OPVD) [45–47], and organic molecular beam deposition (OMBD) [44]. In particular, thermal sublimated deposition is the most popular method for the fabrication of small molecule OPV in vacuum chambers with the pressure about 10^−6^~10^−7^ torr. Generally, tungsten boats and quartz cells are utilized to load and evaporate inorganic and organic material, respectively. High vacuum pressure is required to prevent damaging contaminants, such as oxygen or water molecules, from interfering in the process of organic thin film deposition. The key parameter, deposition rate, is monitored by a quartz crystal microbalance monitors and can be controlled by the heating temperature of the thermal source and used to decide film thickness. By this method, the layer donor, acceptor, EBL, and cathode are deposited sequentially on the anode substrate to form the OPVs. The devices are usually transferred to the glovebox in a 99.95% nitrogen atmosphere and encapsulated to prevent the damage from oxygen or water molecules.

**Figure 3:**
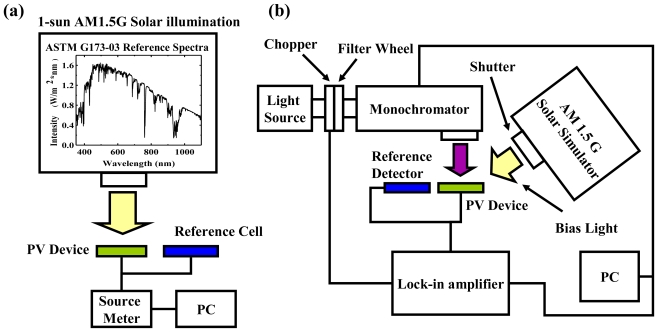
Schematic of (a) the power conversion efficiency (PCE) measurement system and (b) the external quantum efficiency (EQE) measurement system. The insert figure shows the spectrum of the 1 sun AM 1.5G standard solar illumination.

**Figure 4:**
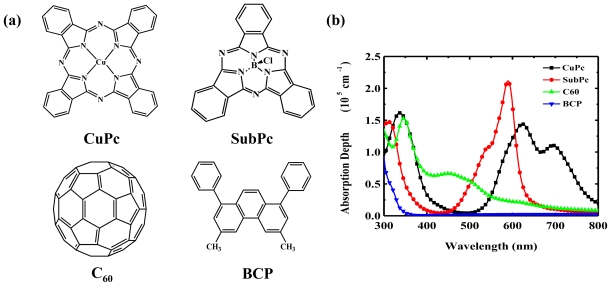
(**a**) Molecular structure and (**b**) absorption spectra of the commonly used small molecule organic materials for OPV devices.

To evaluate the quality of OPV devices, the above two parameters PCE (η_P_) and EQE (η_ext_) in Equations 1 and 2 are needed for the efficiency of power and carrier generation to be measured out individually, by different measurement setups, as shown in Figures 3 and 4. These standard measurement criteria of PCE and EQE passed by American Society for Testing and Materials are required to evaluate the performance of OPV devices in different labs, and avoid misleading results [48]. Figure 3(a) shows the setup of the PCE measurement system. The device is illuminated by a simulated solar spectrum provided by a solar simulator, and the output light power is calibrated through a reference silicon cell. A source meter is employed to provide simultaneous measurements of the voltage and the photocurrent extracted from the device. The *J*-*V* curve can be plotted by scanning different voltages, as in Figure 2(a); and the PCE can be calculated by Equation 2.

The accuracy of the measurement is influenced by the stability of the light source, the integrity of the measurement environment, and the reliability of the devices. To eliminate uncertainty, a number of parameters are strictly formulated. For example, one sun is defined as the standard power 100 mW/cm^2^ and the solar spectrum AM 1.5G, as in the insert of Figure 3 for the measurement criteria [49]. The class of the solar simulator is used to distinguish the difference in spectra between the simulated light and the AM 1.5G [50]. Variations in the measurement environment caused by temperature or humidity may lead to misestimation of the device performance; particularly for organic materials due to its sensitivity to heat, oxygen, and water molecules. Generally, it is recommended that the temperature of the devices be maintained at room temperature (25 °C) with variations of ±0.5 °C, by the temperature-control plane during measurement [51].

EQE is different from the measurement of PCE. It is sometimes called incidental photon to electron conversion efficiency (IPCE), indicating the spectral response of the PV devices. IPCE measurement systems generally consist of a solar simulator, a monochromator with a frequency chopper, and a lock-in amplifier as shown in Figure 3(b) [52]. For measuring EQE spectra, an AM 1.5G solar simulator provides the power of 0.3 to 0.5 suns as the bias light, which is set as the operation point of the device. Photons with specific wavelengths are filtered by monochromator, modulated by chopper with a fixed frequency, and then shone into the OPV device to generate the modulated signal. The signals can be read out using a lock-in amplifier and calculated as the number of photo-generated carriers. The scanning wavelength is generally located within the absorption range of the active materials. Finally, the EQE spectrum can be calculated by the ratio of the number of photo-generated carriers to the number of the incidental photons.

## 4. Efficiency Improvement Techniques

The first efficient small molecule bilayer OPV device comprised an indium tin oxide (ITO) anode, CuPc donor, 3,4,9,10-perylene tetracarboxylic bisbenzimidazole (PTCBI, PV) acceptor and Ag cathode [5], which became a reference structure similar to that shown in Figure 1. There was a planar heterojunction interface between donor and acceptor to benefit the efficient dissociation of the photo-generated excitons. Donor and acceptor provide a homogeneous transport pathway to facilitate the hole, electron transport, and collection. These structural designs and material properties greatly improve the efficiency of the OPV device. Nonetheless, compared with inorganic materials, several intrinsic drawbacks of the organic materials retard the speed of developing high efficiency OPV devices; with regard to issues such as, short diffusion length, low carrier mobility, and poor interface physical properties, etc. Hence, a great deal of effort has gone into ameliorating these material drawbacks to improve the performance of the devices by implementing a number of modifications, designs and improvements in structure, material, photon absorption, carrier injection and transport, as discussed in the following.

### 4.1. Electrode Modifications

The anode and cathode directly influence the built-in potential within the OPV devices, due to the difference of work function between them [53,54]. The variation in work function of a specific electrode also changes the built-in electrical field and even affects the *V*_OC_ of the device [55,56]. OPV devices of different electrode materials with the same work function provide distinct results, due to differences in the polarity of the metal [28]. These effects of work function and the material of the electrodes could be obviated by employing a buffer layer or EBL between electrode and the active layer [57–60]. For example, the poly(3,4-ethylenedioxythiophene):poly(styrenesulfonate) (PEDOT:PSS) acts as the buffer hole transporting layer inserted between ITO and CuPc, as well as BCP acting as the EBL to separate cathode and C_60_ [31]. These options will be discussed in detail later.

For anode applications, ITO on a glass substrate is a common usage due to its high transmittance in the visible range. However, ITO still has several disadvantages such as: Low conductivity (about 100 times lower compared to metals such as Ag and Al) [61–63]; lack of flexibility and breakability [64]; concern about the scarcity of indium [65]; and the migration of indium into the active layer deteriorating the performance of the device [66,67]. Several other substitute conducting materials have been developed for application as the anode in organic electronic devices. These include transparent conducting oxide (TCO) [68–70], conducting polymers [71], carbon nanotubes [72–75], and grapheme [26,76]. For example, Schulze *et al.* used aluminum doped zinc oxides (AZO) as the anode for small molecule OPV devices to achieve a PCE close to 3% [68,69]. However, these substitute anode solutions are still immature and have a number of disadvantages, such as high sheet resistance, a high degree of surface roughness, or difficulties in production. They require additional time and effort to overcome these deficiencies to make them suitable for OPV applications.

Recently, an ITO- and PEDOT:PSS-free top-illuminated device was proposed, featuring a reflective metal anode and a thin metal film as the semitransparent cathode for receiving photons [77–80]. In such inverted structures with all metallic electrodes, surface modification of the anode and the continued transporting pathway of the semi-transparent cathode are critical issues for device performances. Tseng *et al.* presented pentacene/C_60_ small molecule OPV devices with Au anode modified by several self-assembled monolayers [77]. Meiss *et al.* demonstrated the ZnPc:C_60_ bulk heterojunction OPV devices with thick Al anode with p-type dopant modification and Al/Ag double metal thin film as the cathode and maximum power conversion efficiency of 2.21% was reported with the optimal structure [79]. We also reported a top-illuminated structure with a thick Ag anode, which was oxidized by UV-ozone surface treatment to form the silver oxide (AgO_X_) interfacial layer and thin Ag single layer as the semi-transparent cathode, accompanied with a capping layer [81]. The capping layer was a thin film of α-naphthylphenylbiphenyl diamine (α-NPB) deposited atop the cathode to confine the Ag film and match the refractive index, thereby drawing more incidental photons into the active layer and increasing absorption of the active materials by approximately 50%. Additionally, this capping layer could be applied to adjust the optical field distribution within the device. This will be discussed later.

### 4.2. Active Layer

Organic semiconductors consist of π-conjugated molecular compounds based on carbon, hydrogen, nitrogen, and oxygen elements for achieving particular electrical and/or optical functions, such as charge transport, absorption and emission properties. Figure 4 shows the molecular structure and absorption spectra of several small molecule organic materials, which are commonly, used as the active material in the OPV devices. These active materials for photovoltaic applications are usually sorted by their function as turn donor, acceptor, and blocking material. Metal-phthalocyanine such as CuPc, ZnPc, and SubPc, are the most popular hole transporting materials, which usually serve as the donor and dominant absorption material in OPV devices. With different core metals, these materials demonstrate different characteristics with regard to absorption spectra, energy level, and even molecular structure. Mutolo *et al.* introduced SubPc as the donor material. Compared to the planar CuPc molecule, SubPc has a nonplanar cone-shaped structure and can be packed in various orientations, resulting in a strong dependence between energy level and deposition conditions [82]. With strong absorption and high HOMO, the SubPc/C_60_ base OPV device produces a PCE of 2.1% with double the *V*_OC_ (0.97V) of CuPc/C_60_ based devices [28]. Salzman *et al.* replaced CuPc with chloroaluminum phthalocyanine (ClAlPc) as the donor to extend the absorption spectrum to near IR, and the PCE of the device reached 2.1% [29]. C_60_ is widely used as acceptor material in devices due to its high electron mobility, long exciton diffusion length, and complementary absorption spectrum relative to that of major donor materials [31,83–88].

### 4.3. Thickness of the Active Layer

**Figure 5:**
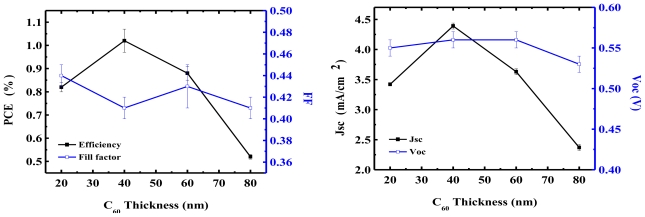
Efficiency parameters of OPV devices with different thicknesses of acceptor material. The devices consist of ITO/CuPc (20 nm)/C_60_ (X)/BCP (7 nm)/Ag (100 nm).

There is a trade-off between low absorption, short exciton diffusion length, and poor carrier mobility of organic materials. Because short exciton diffusion length and poor carrier mobility limit the total thickness of active layers in OPV devices, one cannot increase the thickness of the active layer to increase absorption. Generally, thickness influences electrical performance and optical field distribution within OPV devices. In this section, we focus on the thickness-dependent electrical performance corresponding to the charge imbalance between electrons and holes. Because native electron and hole mobility are usually quite different, whether in the acceptor or donor material, this mismatch results in a charge imbalance, which could be alleviated or varied by adjusting the thickness. Figure 5 shows the performance of the device *versus* the ratio of the thickness of the donor and acceptor in the CuPc/C_60_ planar heterojunction OPV device. The results indicate that the electrical characteristics of the device are entirely different with various thickness ratios. By changing the thickness of the active materials, the optical field in the device is redistributed. This changes the position of absorption peak, resulting in an obvious change in *J*_SC_, which influences *V*_OC_, as in Equation 4. The *J*_SC_ varies from 3.4 to 4.4 mA/cm^2^ while the thickness of C_60_ changes from 20 to 40 nm, respectively. With 80 nm C_60_, *J*_SC_ decreases to 2.37 mA/cm^2^. The PCE changes from 0.82% (20 nm C_60_) to 1.02% (40 nm C_60_), and then decreases to 0.52% (80 nm C_60_). The fill factor does not change obviously, due to the high electron mobility of C_60_, which does not result in the charge imbalance condition of the device. Furthermore, Heutz *et al.* presented similar results for the thickness of CuPc in the ITO/CuPc(x nm)/C_60_ (40 nm)/BCP (12 nm)/Al OPV devices [89]. By varying the thickness of CuPc from 15 nm to 30 nm, PCE was improved from 0.75% to 0.94% and then decreased to 0.67% when the thickness of CuPc was increased to 45 nm.

### 4.4. Injection and Transporting Layer

The injection and transport mechanism could be explained as the carriers jumping from one layer to a neighboring layer and moving forward by one layer. From the above discussion on materials, the donor metal-phthalocyanine is the hole transporting material and dominant absorber of the active layer within the OPV device. It is difficult to balance the energy level alignment between the work function of the anode and the HOMO of the donor, which impedes the hole injection from anode to donor. For example, the energy barrier between the untreated ITO anode and CuPc (SubPc) is approximately 0.3 eV (0.9 eV). Generally, this energy barrier could be reduced by increasing the work function of ITO anode by treating it with plasma [16,90] or UV-ozone [91,92], which is referred to as surface modification. However, the lifted work function of ITO would decay with time and degrade device performance. Therefore, the hole injection layers are applied for the purpose of lowering the energy barrier while the hole transports between the anode and donor. The poly[3,4-(ethylenedioxy)- thiophene]:poly(styrene sulfonate) (PEDOT:PSS) is the most famous hole injection material, which is commonly utilized to decrease surface roughness [31] and increase work function, thereby obtaining an ohmic contact between the anode and donor, increase the hole collection, enlarge the *V*_OC_ [93,94], and block leakage of electrons and excitons from the anode [95,96].

**Figure 6:**
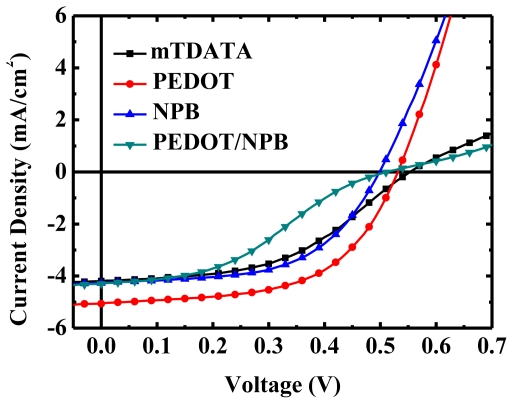
*J*-*V* characteristics of the OPV device with different hole transporting (or injection) layer. The devices consist of ITO/HTL (20 nm)/CuPc (20 nm)/C_60_ (40 nm)/BCP (7 nm)/Ag (100 nm).

We adopted the 4,4′,4″-tris[*N*,(3-methylphenyl)-*N*-phenylamino]-triphenylamine (m-TDATA), NPB and PEDOT:PSS for the hole injection and transport layer for fabrication of OPV devices. The *J*-*V* characteristics of the devices with these materials are shown in Figure 6. The device with PEDOT:PSS as the hole injection layer shows improved performance in the *V*_OC_, *J*_SC_ and *FF*, owing to the increased conductivity of PEDOT:PSS compared to the organic material, which decreases the *R*_S_ and thus increases the *J*_SC_ and *FF*. Furthermore, PEDOT:PSS provides improved energy level alignment with the donor to reduce the energy loss between anode and donor. Decreased energy loss results in a further enhancement of *J*_SC_ and *V*_OC_. Although PEDOT:PSS is quite an effective hole injection material, it remains an issue for the usage of PEDOT:PSS, for example, narrow process window [57,59], etch of ITO due to its intrinsic acidic characteristics, and water absorbance, which deteriorates the device performance [65,97].

To improve performance, the search for a new material to replace PEDOT:PSS. Metal oxide is currently being widely discussed and could be an effective alternative for hole injection material in OPV devices. For example, Shrotriya *et al.* demonstrated the improvement in efficiency of the bulk heterojunction OPV devices by inserting molybdenum oxide (MoO_3_) as the interlayer between the anode and active layer, to obtain a PCE 3.33% higher than devices using PEDOT:PSS [98]. A similar result was reported by Irwin *et al.* with a p-type nickel oxide (NiO) interlayer with a high PCE of 5.16%. The NiO modified the work function of the anode and helped to prevent unexpected chemical reactions between ITO and the active layer [59]. Li *et al.* used SubPc, referred to as a blocking layer, similar to the injection layer of MoO_3_ inserted between SnPc and ITO. The blocking layer helped the SnPc/C_60_ planar structure OPV device to attain a PCE of 2.1%, which was much higher than the PCE of 0.45% for the device without any blocker [24]. Recently, Chauhan *et al.* also presented an interfacial modification by introducing MoO_x_ and 3,4,9,10-perylene tetracarboxylic acid (PTCDA) interlayer as the hole injection double layer. The OPV device based on ITO/MoO_X_/PTCDA/ClAlPc/C_60_/BCP/Al planar heterojunction structure showed a PCE of 3% [25].

Notably, a number of hole transporting materials could serve as a second donor in the OPV devices. Those materials were applied to energy alignment and even contributed to the complementary absorption spectrum for the active layer. This double donor and single acceptor cascade structure possesses the advantages of better energy level alignment, multi-charge dissociation interfaces, and broadband absorption of solar spectrum. For example, Sista *et al.* constructed an OPV device with a cascade-type energy band structure, by inserting CuPc as the interlayer between the donor and acceptor to enlarge the *V*_OC_ from 0.31 V to 0.558 V and improve the PCE from 0.78% to 1.54% [19]. Zhang *et al.* reported another cascade energy level alignment structure using m-TDATA as the hole transporting layer inserted between CuPc and ITO. This structure provided improved energy level alignment and two interfaces for exciton dissociation (m-TDATA/CuPC and CuPC/C_60_) to contribute more than a 30% enhancement in PCE [22]. Furthermore, Yang *et al.* demonstrated an OPV device with a CuPc/tin(II)-phthalocyanine (SnPc):C_60_ mixture/C_60_ cascade structure and broadband absorption to obtain a PCE of 2.9%, because the SnPc had extended the absorption spectrum of the active layer to the near infrared [21].

### 4.5. Exciton Blocking Layer

**Figure 7:**
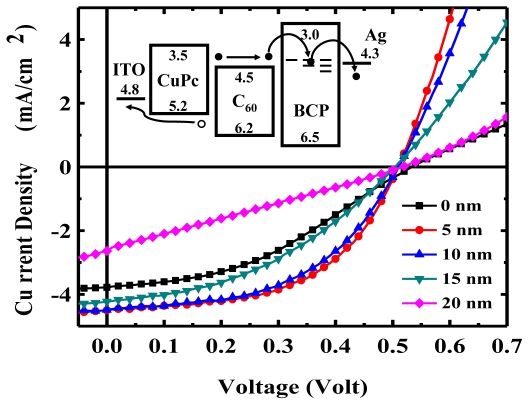
*J*-*V* characteristics of the OPV device with different thickness of BCP layer. The devices consist of ITO/CuPc (20 nm)/C_60_ (40 nm)/BCP (x nm)/Ag (100 nm).

The EBL was first proposed by Peumans *et al.* [9], and became the most famous structure in small molecule OPV devices with the energy level diagram, as shown in the insert of Figure 7. The most popular EBL is BCP [37,43] serving as the buffer layer and inserted to separate the acceptor C_60_ and Ag cathode for the prevention of thermal damage during the deposition of high temperature metal atoms [31,37,38], and the exciton quenching induced by the cathode [9,35]. The usage of BCP prohibits electron transfer from metal to C_60_ and prevents an increase in work function at the C_60_/metal interface, thereby maintaining the built-in electrical field in a steady state as a benefit to the collection of carriers [35,37,99]. For example, in Figure 7, we illustrate several OPVs based on CuPc/C_60_ with BCP of various thicknesses as the blocking layer for the estimation of JV performance. The *J*_SC_ performance of the devices can be easily enlarged by inserting an ultra thin BCP layer (5~10 nm) to separate the C_60_ from the cathode. Moreover, the BCP is not only employed in small molecular OPV devices, but can be found in conjugate polymer OPV devices [60]. Typically, qualified EBL materials possess wide band gap and high electron mobility, while offering good thermal stability. The wide band gap above the energy level of the acceptor material cannot be absorbed and remains transparent for the absorption range of the active layer. It also prevents dissociation of the exciton at the interface between the acceptor and EBL. The strong electron transporting properties retain carrier collection ability and suppress the *R*_S_ of the device. Finally, good thermal stability is helpful to extend the lifespan of the device [42,100,101].

However, the low carrier mobility of BCP and the alignment mismatch of the energy level between the LUMO of BCP, and the work function of the cathode impede the carrier collection and injection. Because of such weaknesses, the thickness of BCP influences the *J*-*V* performance of OPV devices, as shown in Figure 7. The device without the BCP layer delivered the poorest performance, particularly with regard to *J*_SC_ and *FF*, due to the thermal damage caused during metal deposition and the exciton quench from the metal cathode. The *J*_SC_, *FF*, and PCE of the devices were considerably improved after introducing the ultra thin BCP as an EBL of less than 10 nm. It was observed that the *R*_SH_ and *J*_SC_ diminished rapidly with an increase in the thickness of BCP layer beyond 10 nm, resulting in a decrease in the PCE of OPV devices. This indicates that the thickness of BCP definitely dominates the electron transport behavior within the device. When the thickness of BCP is increased, the transported electrons are easy to collect and recombine in the acceptor layer near the acceptor/BCP interface due to the poor electron mobility of BCP, thereby decreasing the *R*_SH_ and *J*_SC_. The increase in *R*_S_ with the thicker BCP thickness results from the integral resistance of the device. The poor stability of BCP caused by the low glass transition temperature (*T*_g_) causes the amorphous film to crystallize easily under high temperature. This is a serious issue for the reliability of the OPV device [89,102].

To ameliorate the thickness dependent problem of BCP, Chen *et al.* employed ytterbium (Yb) doped bathophenanthroline (Bphen) to increase the electron mobility and reach improved energy level alignment between the EBL and the cathode, resulting in constant electrical properties when the EBL thickness was increased from 5 nm to 40 nm [36]. Rand *et al.* replaced BCP with tris(acetylacetonato) ruthenium(III) (Ru(acac)_3_) to improve hole injection under reverse bias. The holes could be injected directly through the HOMO of the Ru(acac)_3_ from the electrode to the active layer better than injection through the defect state of the BCP [33]. The devices showed nearly identical electrical properties when the thickness of Ru(acac)_3_ was increased to 30 nm. These results show the ability of EBL materials to act as optical spacers with thickness independent characteristics.

To improve the reliability of the devices, Song *et al.* used reliable tris-8-hydroxy-quinolinato aluminum (Alq_3_) to replace BCP as the EBL, as a blocking layer against the diffusion of the metal atoms from the cathode and permeation of oxygen and water molecules. In this regard, it lengthened the device lifetime drastically, by more than 20× compared to the device with BCP [34]. Furthermore, Liu *et al.* demonstrated the ball-like material 4-hydroxy-8-methyl-1,5-naphthyridine aluminum chelate (AlmND_3_) as an EBL with a better device lifetime, compared with the device using BCP as the EBL [103]. This new material, AlmND_3_, exhibits several advanced characteristics such as wide band gap (3.3 eV), high electron mobility (~10^−4^ cm^2^ V^−1^s^−1^ at the electric field of 6.4 × 10^5^ Vcm^−1^), and high glass transition temperature (~194 °C) compared to those of BCP. Unlike Alq_3_, OPV devices produced with AlmND_3_ as the EBL showed an increase in photovoltaic performance owing to its relatively high electron mobility, which maintained the electron collection efficiency of the device, resulting in higher *J*_SC_ and *FF* than OPV devices produced with Alq_3_ as the EBL. In addition, the high glass transition temperature of AlmND_3_ resulted in a more stable surface morphology than that of BCP at elevated temperatures. Hence, the characteristics of AlmND_3_ were shown to prolong the lifetime of OPV device based on Pentacene/C_60_, without compromising efficiency.

### 4.6. Internal Optics

**Figure 8:**
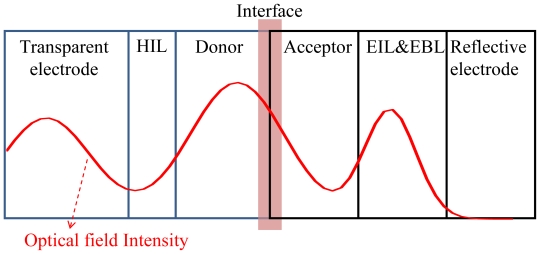
Distribution of optical field intensity inside a complete OPV structure with two electrodes, injection layers for hole and electron, blocking layer for excitons and a bilayer donor/acceptor.

According the above discussion, *V*_OC_, *FF* and *J*_SC_ are the important factors determining the PCE of OPV devices under solar illumination. In particular, photocurrent *J*_SC_ is strongly dependent on the absorption ability of the active layer and the optical field distribution within the device. Absorption ability is material dependent and optical field distribution is device structure dependent. In Figure 8, the optical field distribution within a multi-layer device is given by the propagation of incident light interfering with the reflection from the reflective electrode [104,105]. The ideal case was achieved when the position of maximum optical field intensity was coincidentally located at the interface of the heterojuction, the main region for exciton dissociation. A great number of photons become excitons and then dissociated at the interface, which is supposed to initiate a stronger photocurrent. Notably, the thickness and optical properties (refractive index: *n*, absorption coefficient: *k*) of each layer function as critical factors to determine the distribution of the optical field [106]. The optical field was calculated using the transfer or scattering matrix for the simulation of amplitude and its location within the OPV device. The key factor determining the amplitude of the distributed optical field is relative to the phase Δφ = 2π[*n*(λ) − i*k*(λ)]*d*/λ, where *d* is the optical path dependent on the thickness of each layer within the OPV device.

According to the above calculations, redistributing the electrical field by fine tuning the thickness of the active layer to optimize the location of the maximum optical field is a simple task [63]. For example, Hur *et al.* fixed the thickness of the CuPc at 20 nm and utilized the thickness of a single layer of C_60_ between 20 and 80 nm in pursuit of the optimal thickness ratio of 2:1 between CuPc and C_60_ to obtain a high PCE [107]. This suggested the presence of a stronger optical field located at the interface of the donor/acceptor in this condition. Lee *et al.* provided more experimental data [108], in which they varied the thickness of CuPc (5–20 nm), C_60_ (5–30 nm), and BCP (1–15 nm) to obtain improvements in the PCE of 330%, 118%, and 112%, respectively, compared to their lowest value. They reported that the thickness of the CuPc was a key factor in determining the PCE of OPV devices, due to its low hole mobility (100× lower than the electron mobility of C_60_). On the other hand, it was implied that the thicknesses of the high electron mobility materials, acceptor C_60_ and blocking layer BCP could be used to adjust the distribution of the optical field. In addition, from these results, a stronger intensity of the optical field located at the interface of the donor/acceptor was not the primary factor in improving PCE. The carrier transport behavior of the donor and acceptor appear to have been more important, and had to be optimized because they were thickness-dependent. In general, a thinner organic layer delivers better carrier transport behavior. However, the thinner active layer in the OPV device resulted in a reduced absorption, leading to a decrease in the generation of excitons. The decrease in exciton generation corresponded directly to the reduced *J*_SC_ and PCE. Hence, there was a tradeoff between the thickness-dependent carrier transport and the absorption of organic materials limiting the degree to which the thickness of the layer could be tuned [109].

Without modifying the thickness of the active layer, several technical reports have emphasized inserting a buffer layer inside OPV [110–117] and adjusting its thickness to arrange the maximum optical field intensity near the interface, to increase PCE. The buffer layer could be the calcium (Ca) [110], tungsten trioxide (WO_3_) [111,115], lithium fluoride (LiF) [113,114] as electron injection layer; or PEDOT:PSS [108], ZnO [111,115], MoO_3_ [110,113] as the hole injection layer; or BCP [108], BPhen [36,118] as exciton blocking layer. They were used not only to separate the active layer from the metal electrode to prevent exciton quenching, but also redistribute the optical electrical field within the device.

For example, Lee *et al.* inserted thick HIL spacers to simulate an optical electrical field of greater intensity confined within the inverted OPV, thereby increasing the *J*_SC_ by up to 52.6% [119]. Without considering the issue of thickness-dependent carrier mobility, applying a capping layer on the OPV device to fine tune the internal optical field was easier by using materials, such as NPB [81] and Alq_3_ [78,117]. Meiss *et al.* showed that using an Alq_3_ capping layer as an index matching material permitted an increase in the coupling of incident light in the device by tailoring the distribution of the optical field. Controlling the optical field distribution and increasing the absorption efficiency of a specific absorbing layer proved to be convenient. PCE could be improved by up to 50% using a 60 nm Alq_3_ capping layer [80]. Chen *et al.* demonstrated the insertion of a transparent ITO in front of the highly reflective Ag cathode to redistribute the spatial optical field within the OPV to increase *J*sc and improve PCE without increasing absorption [120]. In particular, Chan *et al.* provided a material, Yb-doped BPhen, combining high transparency with good electrical conductivity to act as an exciton blocking layer and optical spacer to increase *J*sc and PCE [36]. This is a strong candidate for the adjustment of the internal optical field.

Another approach to adjust the optical field is the implementation of two or more devices comprising several layers operating in tandem; however, this approach is somewhat complicated. One of the layers or devices is adjusted to optimize the optical intensity at the interface of the donor/acceptor. As reported by Xue *et al.* stacked OPV devices showed the greatest intensity of long wavelength optical field occurring at the front of the cell, with the greatest intensity of short wavelength optical field located at the back of the cell. A maximum PCE of 5.7% was achieved by combining the optimal contribution from the front and back of the cells [121]. Drechsel *et al.* used high transport materials (p-type MeO-TPD and n-type C_60_) to control the distance of the center of the two stacked OPV devices, leading to a rearrangement of the optical field. The peaks of simulated absorption flux were located in the active layers to generate more excitons [13]. Schueppel *et al.* also simulated spacers of several thicknesses (0–186 nm), using p-type transparent material, inserted between two tandem cells to optimize the absorption peaks located near the active layer for the generation of more excitons [122].

Replacing one or more layer materials with materials possessing suitable optical properties has also been considered to modify the distribution of the optical field. Long worked on simulating three electrode metals Al, Au, and Ag to rearrange the optical field distribution inside an equivalent organic configuration [111]. However, it was difficult to obtain the materials with the expected *n k*, which also contained the qualified electrical characteristics and fabrication requirements for application in high efficiency OPV devices. A well-known approach to alter the *n k* of materials by doping or mixing with other materials is easily achieved, as reported by Sarasqueta *et al.* [123]. He used Ag dopant to modify the *n k* value of the organic host. For organic dopant, the 50% rubrene [10] and 4% pentance [124] doped in CuPc layer of OPV device was reported to obtain a significant improvement in PCE 2.13 and 1.77 times, respectively. The reason is not only the rearrangement of optical field distribution, but rubrene and pentance dopants contribute complementary absorption spectra and enhanced carrier mobility for CuPc host.

Currently, the concept of complementary functions to modify the characteristics of layers by doping is hot topic; particularly for boosting efficiency. The absorption ability of OPV devices, the surface plasmonic resonance (SPR) and local (L−) SPR absorption generated from nanoparticles have been employed to facilitate the harvesting of photons inside devices [125,126]. In fact, SPR and LSPR are mechanisms of energy transformation, in which incident optical waves are transformed into electromagnetic waves propagating along the metal/organic interface [127]. This transformation is induced by the formation of a stronger electrical field near the metal particles of nano-dimensions coupling with the incident optical electrical field, whereupon it assumes another resonant frequency to travel around it. After some traveling time, the incident energy is absorbed or transformed to become nonradiative waves or another resonant frequency through nanoparticle or organic matrix, namely SPR and LSPR absorption.

**Figure 9:**
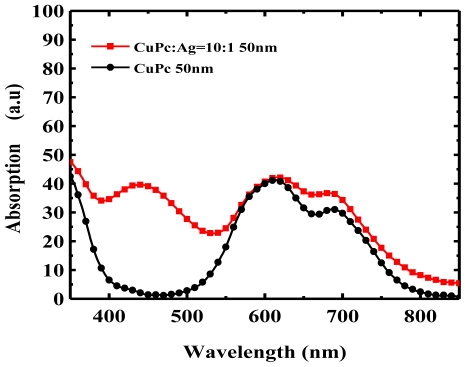
Comparison of absorption spectra between CuPc and CuPc doped with Ag nanoparticles.

**Figure 10:**
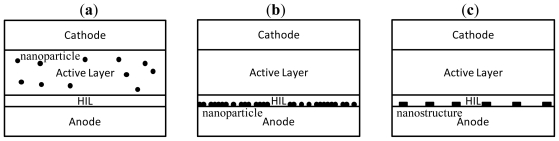
Plasmonic effects from (**a**) nanoparticles mixed in active layer and (**b**) HIL, (**c**) nanostructure on anode.

According to the Mie theory [128], SPR effects are material dependent and the absorption spectra are determined by the size and shape of the nanoparticles [129,130]. Suggested materials include Ag [131], Au [132], ZnO [133], zinc sulfide (ZnS) [134], cadmium selenide (CdSe) [135], and the shapes could be circular, triangular [136,137], rectangular [47], pillar shaped [138], *etc*. [139]. In Figure 9, we show the SPR and LSPR absorption from Ag nanoparticles to compensate for the deficient absorption spectra of CuPc thin film near 450 nm. The doping ratio between CuPc and Ag nanoparticles is 10/1 by volume ratio. Moreover, the SPR absorption spectrum could be controlled by adjusting the doping ratio and due to the larger size of the nanoparticles could tolerate a higher doping ratio [131]. For device applications, such as those proposed by Kim *et al.* [140], Ag and Au nanoparticles doped in the mixture active layer show a 50 to 70% improvement in PCE, as shown in Figure 10(a). The mechanism of embedding metal nanoparticles into PEDOT:PSS, as shown in Figure 10(b), was a more popular method of enhancing absorption and PCE [125,126]. Morfa *et al.* employed a thin plasmonic layer formed by a thin Ag film of 1 nm to obtain a significant improvement in PCE of 1.7-fold [125].

Moreover, Lee *et al.* also used the Mie approach to simulate the combined absorption of spherical metal nanoparticles in the active layer, mentioning that it resulted mainly from a stronger scattering effect. Enhanced optical absorption was localized, induced by a stronger local optical electrical field scattering the incident photons near the nanoparticles [141]. Kim *et al.* also utilized non-absorptive ZnO nanoparticles embedded in PEDOT:PSS and closed to anode to scatter incident photons resulting in an enhancement of optical absorption, leading to an increase in *J*_SC_ [142]. In addition, Fahr *et al.* used Ag nanoparticles embedded in the ZnO layer to induce scattering, resulting in strong plasmonic absorption to enhance PCE [143].

Additionally, the nanostructures in Figure 10(c) also contribute to the SPR absorption, referred to as periodical dimension and shape [47,144,145]. These are widely employed for upgrading the PCE of OPV devices. Lindquist *et al.* used an Ag nanostripe as a patterned anode with a period of 409 nm and a slit width of 120 nm (like a grating) to obtain a 3.2-fold increase in PCE, compared with an unpatterned Ag anode [146]. Min *et al.* reported similar results in which the nanostripe grating resulted in the enhancement of broadband absorption for incident p-polarized light. The overall enhancement of absorption was as high as 50% [147]. Several nanostripes were formed using Au [148] and low refractive index conducting materials [149] to report the enhanced SPR absorption. These nanostripe electrodes were substituted with transparent metals such as ITO, Au and Ag, nanoimprinted to control their period and physical pitch [150]. Hence, it is easier to obtain a pattern electrode and combine with other effects to raise the OPV performance like Zou *et al.* used the Ag stripe and ZnO nanoparticle at the same time to contribute the PCE enhancement [151].

Dissimilar to nanostripe, Bai *et al.* proposed an alternative, in which periodic nano-hole structures were patterned in an Ag cathode to induce surface plasmonic absorption within the OPV device. By carefully controlling the physical size and periodical pitch of the nano-holes, they increased the absorption of OPV from 39% to 112%, corresponding to an increase in *J*_SC_ from 47% to 130% [152]. Atwater *et al.* described nanostructures of various shapes: Hexagonal arrays of Ag nanoparticles, arrays of coaxial holes in a metal film; and antenna arrays to couple incident light into the OPV device to trap light and increase absorption through plasmonic effects [153,154].

### 4.7. External Optics

To improve the PCE of OPV devices without modifying the internal structure, external optical engineering is required to increase the light coupled into the device or effectively exhaust the incident light. Such external optical engineering would involve extra optical components such as microlenses, OPV devices, or structures for optical confinement. A common commercial approach has been to apply a microlense for coupling the light out of the emitting medium. It has also been beneficial to couple incident solar light into the OPV device to increase light flux, leading to an increase in *J*_SC_. The focal length of each lens could be configured to focus near the interface of the donor/acceptor to increase the probability of exciton dissociation. This microlense could also be configured to combine the trapped light to increase the *J*_SC_ by as much as 25%, as reported by Zilio *et al*. [155,156]. In their study, the light trapping structure was a Fabry-Perot resonant cavity with many apertures precisely created from an array of aligned microlenses. Most of the photons entered the cavity through these apertures, recycling reflections within it. In this manner, the light path was extended to increase absorption.

However, it is difficult to completely consume incidental light by a round-trip optical path inside an OPV device, even with the above auxiliary absorption mechanisms [157] or though two or more devices operating in tandem [122]. Two or more OPV devices operating in tandem is a straightforward approach to increasing absorption, due to process compatibility, such as two or more OPV devices work at the same time. Nonetheless, stacked OPV devices still have difficulty completely absorbing the incident light due to optical loss from surface reflection. Unlike a stacked configuration, Rim *et al.* used two OPV cells to construct a V-shape waveguide with an angled opening. They controlled the angle of the opening to evaluate how many times the light could be reflected in this waveguide. An increase in the number of reflection times contributed directly to an increase in absorption and *J*_SC_. Two 0.81 mm^2^ OPV devices with a 35° opening angle increased *J*_SC_ by 52% [158]. Similar results were reported by Tvingstedt *et al.*, who employed two different OPV devices specified for two different absorption bands to form a simple geometrical V-shape waveguide. One device absorbed the non-absorptive spectrum reflected from the other to enhance absorption, leading to an increase in PCE [159].

**Figure 11:**
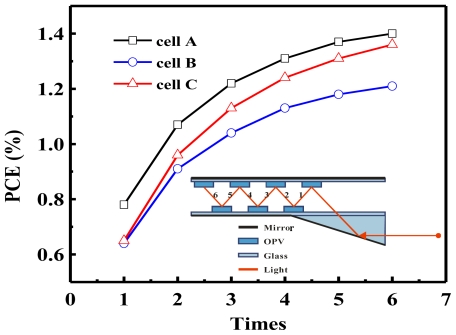
Efficacy enhancement after few reflections for device A, B and C with different PCE values and reflectance in a multiple reflection cavity system.

In order to efficiently exhaust incident solar light, a great many external optical designs based on multiple absorption have been discussed. These include light harvesting waveguides [155,160], or multiple reflection cavity systems [158], to trap photons until absorbed by a series of OPV devices or an OPV module, as shown in the inset of Figure 11. The optical design applied for dye sensitizer solar cells could improve the PCE by 4.3 times (final: 0.54%) [160]. We employed the small molecular planar heterojunction inverted OPV devices into this optical system to obtain the obvious enhancement of final PCE. The reflectance and EQE of device are key factor to affect the final results. Because the inverted OPV device is a cavity structure, the thickness of interlayer affects the reflectance and EQE. Hence, we used device A, B and C with different active layer (CuPc/C60: 15/40, 15/30 and 20/30 nm). In visible range, their average reflectances are 40.9, 43.8 and 47.8%, and their average EQE are 15.9, 15.36 and 15.43% in turn. The device has a higher degree of reflectance in this light trapped system showing a 2.09 times increase in PCE after 6-time reflections. In this experiment, we prove that the initial high PCE in the device does not necessarily indicate that this light trapped system would be optimal. One key factor that still requires attention is to find the balance point between reflectance and PCE of OPV devices.

## 5. Conclusions

In summary, we have reviewed several approaches to achieve high efficiency OPV devices with metal-phthalocyanine/C_60_ active layer structure. The efficiency could be improved by engineering device architecture to eliminate carrier-transporting obstacles, reduce carrier-injection barrier, avoid exciton quenching, increase incident photon numbers and absorption, and rearrange internal optical field distribution, *etc*. It is possible to improve most of the devices by inserting a hole injection layer, PEDOT:PSS or metal oxide, to obtain a high PCE of over 3%. In addition, the nanoparticles, dopants and layers were integrated into the device structure as photon absorbers to increase the exciton number resulted in more carrier generation. The tandem OPV device is capable of absorbing more incident photons to improve PCE by more than 5.7%. However, the incident light power from light source is still hard to exhaust by a round-trip path in an OPV device. The external optical designs for multiple reflections are effectively utilized to deplete the incident photons by OPV devices to obtain double the PCE enhancement. Ideally, a PCE greater than 10% could be achieved using internal optical engineering to optimize the individual units in the tandem device and employing this tandem device as a multiple reflection structure. Hence, we believe that the efficiency of OPV device will soon be increased to a level at which they are commercially viable.

## Figures and Tables

**Table 1:** The device structures and the efficiencies (under 100 mW/cm^2^ AM 1.5G illumination) of the metal-phthalocyanine/C_60_ based small molecule OPV devices with planar structure. The layer structure of the control devices in each reference are ITO/CuPc (or ZnPc)/C_60_/BCP (or Bphen)/Al (or Ag) with different thicknesses.

Year	Modifications	Device Structure	Maximum PCE (%)	PCE of Control Device (%)	Ref.
2008	anode material	graphene/CuPc/C_60_/BCP/Ag	0.4 (85 mW/cm^2^)	0.84	[26]
2009		AZO/CuPc/C_60_/TPBI/Al	1.30	1.1	[27]
2005	anode modification	ITO/H_3_PO_4_/ZnPc/C_60_/BCP/Al	1.70	1.20	[15]
2006		ITO/CuPc/C60/BCP/Al	1.90	1.90	[16]
2006	donor material	ITO/SubPc/C_60_/BCP/Al	2.10	1.20	[28]
2007		ITO/ClAlPc/C_60_/BCP/Ag	2.10	1.80	[29]
2010		Mg:Al/C60/SubPc/MoO_3_/ITO	2.40	-	[30]
2010		ITO/ ClAlPc/C_60_/BCP/Al	1.8	-	[25]
2006	acceptor material	ITO/PEDOT:PSS/CuPc/PCBM/BCP/Al	1.18	0.77	[32]
2005	multi-heterojunction	ITO/CuPc/SnPc/C_60_/BCP/Ag	1.00	-	[17]
2007		ITO/F_4_-TCNQ/ZnPc/(C_60_/ZnPc)*3/Bphen/Al	2.20	1.30	[18]
2007		ITO/PEDOT:PSS/TT/CuPc/C_60_/BCP/Al	1.54	1.17	[19]
2007		ITO/ZnPc/PbPc/C_60_/Al	1.95	1.00 (wo EBL)	[20]
2008		ITO/CuPc/C_60_/SnPc/C_60_/BCP/Ag	2.90	-	[21]
2009		ITO/m-TDATA/CuPc/C_60_/BCP/LiF/Al	0.72 (20 mW/cm^2^)	0.54 (20 mW/cm^2^)	[22]
2009		ITO/CuPc/SubPc/C_60_/Bphen/Al	1.29 (80 mW/cm^2^)	0.64 (80 mW/cm^2^)	[23]
2009		ITO/SubPc/SnPc/C_60_/BCP/Al	2.10	-	[24]
2010		ITO/MoO_X_/PTCDA/ClAlPc/C_60_/BCP/Al	3.00	-	[25]
2005	EBL	ITO/CuPc/C_60_/Ru(acac)_3_/Ag	2.7	1.1	[33]
2005		ITO/CuPc/C_60_/Alq_3_/Al	2.11 (75 mW/cm^2^)	1.39 (75 mW/cm^2^)	[34]
2006		ITO/PEDOT:PSS/ZnPc/C_60_/BCP/Al	1.50	0 (wo EBL)	[35]
2006		ITO/CuPc/C_60_/Bphen:Yb/Al	3.42	2.64	[36]
2009		ITO/SubPc/C_60_/BCP/Al	3.03	0.05 (wo EBL)	[37]
